# GnRH Immunocastration in Male Xizang Sheep: Impacts on Rumen Microbiome and Metabolite Profiles for Enhanced Health and Productivity

**DOI:** 10.3390/ani14202942

**Published:** 2024-10-11

**Authors:** Xiaoming Zhang, Tianzeng Song, Guiqiong Liu, Jing Wu, Yangzong Zhaxi, Shehr Bano Mustafa, Khuram Shahzad, Xiaoying Chen, Wangsheng Zhao, Xunping Jiang

**Affiliations:** 1 Institute of Animal Science, Xizang Academy of Agricultural and Animal Husbandry Science, Lhasa 850009, China; 18981473583@163.com (X.Z.); songtianzeng123@sina.com (T.S.); lpzhangyang@163.com (Y.Z.); xzxmsyxh@126.com (X.C.); 2 College of Life Sciences and Engineering, Southwest University of Science and Technology, Mianyang 621000, China; wj15196278568@163.com (J.W.); shehrmustafa@gmail.com (S.B.M.); 3 College of Animal Science and Technology, Huazhong Agricultural University, Wuhan 430070, China; liuguiqiong@mail.hzau.edu.cn; 4 Department of Biosciences, COMSATS University Islamabad, Park Road, Islamabad 45550, Pakistan; khuramsb@gmail.com

**Keywords:** male Xizang sheep, GnRH immunocastration, rumen, microorganisms

## Abstract

**Simple Summary:**

Castration is a routine procedure in sheep farming, and immunocastration is beginning to be used in animal production as an alternative to surgical castration. Currently, there are few studies on the intra-rumen environment after immunocastration in sheep. Therefore, in this study, male Xizang sheep were selected for surgical castration and GnRH immunocastration treatment, the intact control group and immunocastrated group were selected in combination with body weight data, and microbiology and metabolomics techniques were used to analyze the effects of GnRH immunocastration on the rumen internal environment of male Xizang sheep. The results showed that GnRH immunocastration had a more obvious effect on body weight gain while achieving the effect of de-population; immunocastration increased the ratio of rumen thick-walled bacterial phylum to anamorphic bacterial phylum, which improved the digestive utilization of forage feed in male Xizang sheep. Secondly, GnRH immunocastration can maintain the balance of the rumen internal environment and the health of rumen epithelial cells by improving the levels of certain metabolites in the rumen.

**Abstract:**

Castration is a prevalent and indispensable practice in sheep husbandry, aiding in enhancing meat quality, mitigating aggressive behavior, and managing unwanted reproduction. Nevertheless, the conventional surgical castration procedure poses several challenges, including heightened stress and pain, detrimental impacts on animal welfare, and diminished economic efficacy in farming operations. Consequently, immunocastration methods, serving as substitutes for surgical castration, are progressively finding application in livestock. The rumen, an essential and distinctive digestive and absorptive organ in ruminants, has been associated with enhanced meat quality and productive performance following castration in previous research studies, albeit fewer investigations have explored the potential impacts of GnRH immunization on the rumen’s internal milieu in sheep post-de-escalation. Hence, the present study delved into evaluating the impact of GnRH immunocastration on the rumen microbiome and metabolomics in male Xizang sheep. This was achieved through the establishment of a GnRH immunocastration animal model and the collection of rumen fluid for microbiological and comprehensive metabolomics investigations. The outcomes of this investigation unveiled that the impact of GnRH immunocastration on body weight gain was more pronounced during the achievement of the castration objective. In addition, the *Firmicutes-to-Bacteroidota* ratio in the immune male (IM) group exceeded that of the control group (EM), suggesting that GnRH immunodeficiency may enhance the digestion and absorption of feed in male Xizang sheep. At the taxonomic level, the elevated presence of *Prevotella* and *Quinella* bacteria in the IM group compared to the EM group indicated that castration influenced a segment of the rumen microbiota in male Xizang sheep, thereby bolstering the digestive and metabolic efficacy of the rumen concerning nutrient utilization, particularly in the breakdown and absorption of proteins, carbohydrates, and lipids, ultimately expediting the fattening process and weight gain in male Xizang sheep following castration. Moreover, analysis of ruminal fluid metabolomics revealed that GnRH immunization had notable impacts on certain metabolites in the ruminal fluid of male Xizang sheep, with metabolites like 5-hydroxyindole acetic acid and 3-hydroxyindole acetic acid showing significant downregulation in the IM group compared to the EM group, while niacin and tyramine exhibited significant upregulation. These findings indicate a profound influence of GnRH immunization on the maintenance of ruminal equilibrium and ruminal health (including the health of ruminal epithelial cells). This study validates that GnRH immunocastration not only achieves the objectives of castration but also enhances ruminal health in male Xizang sheep, thus laying a foundational theoretical basis for the application and dissemination of GnRH immunocastration technology.

## 1. Introduction

The number of sheep in Tibet exceeds 50 million [1], thereby granting Tibetan herders access to a plethora of resources including meat, milk, fuel, and fur. This breed predominantly inhabits the Tibetan Plateau and its adjacent territories (at elevations ranging from 2500 m to 4500 m) and demonstrates remarkable adaptation to the harsh environmental conditions of the plateau, characterized by high altitude, low oxygen levels, and cold temperatures [2]. The castration of rams serves as a standard and essential practice in sheep breeding management to enhance meat quality, curb aggressive tendencies, and manage unwanted reproduction [3]. Nevertheless, the conventional surgical castration method is associated with issues including labor intensity, complex surgical procedures, susceptibility of surgical wounds to infections, and a propensity to induce considerable stress in animals, leading to heightened stress levels, pain, compromised animal welfare, and decreased economic efficiency in breeding practices [4,5]. Consequently, the adoption of the immunocastration method, serving as a viable alternative to surgical castration, is progressively gaining traction in animal practices. Immunocastration, as an animal-friendly and economically feasible castration approach, offers the advantage of mitigating the adverse consequences associated with surgical and rubber band castration, all the while enhancing meat quality and growth performance [4,6]. Immunization targeting gonadotropin-releasing hormone (GnRH), commonly referred to as immunocastration, has been studied in male rats, Holstein bulls, pigs, and various other animals, revealing substantial enhancements in both meat quality and growth performance, concurrently with the effective suppression of animal reproductive activities [7,8,9]. Generally, immunocastration is effective, safe, animal-friendly, and suitable for animals of all ages, so it can be the best alternative to other castration techniques [4].

The rumen functions as a distinct digestive organ in ruminants, housing a complex and extensive microbial ecosystem. Through the collective activities of bacteria, fungi, protozoa, and other microorganisms, approximately 75% of dietary fibers are digested and transformed into utilizable compounds like protein and volatile fatty acids [10,11]. Yao G et al. [12] observed that castrated boars exhibited a distinct gut microbial composition, characterized by an elevated presence of Firmicutes and Bacteroidetes in comparison to intact boars. Li et al. [13] demonstrated that castration induced alterations in both the composition and functionality of cecal microorganisms in Holstein bulls, resulting in a reduction in the relative abundance of bacteria. Therefore, in the present study, selected male Xizang sheep were divided into intact control, surgical castration, and GnRH-immunocastrated groups for a 112-day feeding trial. Finally, rumen fluid from intact control and GnRH-immunocastrated groups were selected for microbiome and metabolomics analysis and comparison according to body weight changes and study objectives as well as limitations of realistic conditions. The aim of this study was to investigate the potential effects of GnRH immunocastration on the rumen internal environment, microbiota, and metabolomics in male Xizang sheep. This study provides basic data and a theoretical basis for in-depth research on the relationship between rumen microbiota and meat quality changes and the use of GnRH immunocontraception in sheep rearing practice.

## 2. Materials and Methods

### 2.1. Ethics Statement

The sample collection protocol received approval from the Livestock and Poultry Breeding Committee of Southwest University of Science and Technology (SWUST), and all animal-related procedures were carried out in strict compliance with SWUST guidelines (Approval No. L2022026-2022-03).

### 2.2. GnRH Vaccine Preparation

Employing the methodology described by Oonk et al. [14] and our previous work [15], a GnRH vaccine was prepared through chemical synthesis. To facilitate conjugation with carrier proteins, a synthetic GnRH decapeptide was assembled by ligating D-lysine at the 6th position, in lieu of glycine, within the GnRH molecule. Post-peptide synthesis, conjugation with ovalbumin (OVA) was carried out, followed by purification using high-performance liquid chromatography. The resulting conjugate was then emulsified with Specol adjuvant to yield the GnRH vaccine.

### 2.3. Experimental Design and Animal Sample Collection

The experimental animals were obtained from Longri Stud Farm, Hongyuan County, Aba Prefecture, Sichuan Province, China. Thirty 6-month-old male Xizang sheep of uniform weight (30 ± 1.5 kg), good health condition, and before sexual maturity were carefully selected. They were randomly divided into control (EM), orchiectomy (ORC), and immunocastrated male (IM) groups, with 10 animals in each group. The experimental procedure was divided into a 14-day acclimatization period and a 112-day formal trial period. On the first day of the adaptation period, sheep in the ORC group were surgically castrated. At the end of the acclimatization period, male Xizang sheep in the IM group were selected to receive a subcutaneous injection of 2 mL of GnRH emulsion antigen (containing 100 µg of TDK) in the neck. The day of injection of GnRH emulsion antigen was recorded as the first day of the official trial period. On day 56 of the official test period, the male Xizang sheep in the IM group were injected again with GnRH emulsion antigen at the same dose and in the same manner as the first immunization; the EM group was not injected with GnRH emulsion antigen and was not castrated during the whole test cycle. All the experimental sheep were free-grazed on the pasture of Longri Ranch, Hongyuan County, Aba Prefecture, Sichuan Province, without any supplemental feeding, from July to November. The main pasture grasses in the pasture included summer grass (*Carex capillifolia*), barnyard grass (*Poa pratensis* L.), Matang grass (*Potentilla anserina* L.), and bare crown chrysanthemum (*Carex nudicarpa*). All sheep were weighed every 28 days during the formal trial. All sheep were fasted one day before the end of the formal trial. After 12 h of fasting, all sheep were weighed, and blood was collected from the jugular vein of five sheep randomly selected from the three groups. Based on the weight gain, purpose of this study, and other limiting factors, EM and IM groups were selected, and according to the ethically approved method, the test sheep were slaughtered to collect rumen chyme (the sheep slaughtered in EM and IM groups were the same as those slaughtered at the time of blood collection), the rumen fluid was filtered through four layers of gauze, extracted and dispensed into 2 mL cryopreservation tubes, and preserved in liquid nitrogen for subsequent measurement of the experimental data.

### 2.4. Live Weight and Serum Testosterone Determination in Sheep

All sheep in the three groups were weighed before morning grazing on days 1, 28, 56, 84, and 112 of the official experimental period. To verify the validity of the castration method used in this study, blood samples were collected from the jugular vein of five randomly selected male Xizang sheep from the EM, ORC, and IM groups, respectively, on day 112 of the formal experimental period (the sheep for subsequent weight data analysis were kept the same as those for blood collection). The blood samples were allowed to stand and then centrifuged at 3000 rpm for 15 min, and the serum was separated and stored at −20 °C. The blood samples were then centrifuged at 3000 rpm for 15 min. Testosterone levels in serum were determined by ELISA using a sheep T ELISA (KIT/96 kit, ZC-51301, purchased from ZCIBIO Technology Co, Shanghai/China). The experimental procedure was carried out in strict accordance with the instructions of the reagent manual.

### 2.5. Rumen Fluid Sample Processing and Metabolite Analysis

The sample stored at −80 °C refrigerator was thawed on ice and vortexed for 10 s. A 200 μL extract solution (ACN/methanol = 1:4, *v*/*v*) containing internal standard was added into 200 μL sample. Then, the sample was vortexed for 3 min and centrifuged at 12,000 rpm for 10 min (4 °C). A 350 μL of the supernatant was collected and totally concentrated. A 150 μL solution (methanol/water = 7:3, *v*/*v*) was used to reconstitute the residual; it was vortexed for 3 min and then sonicated for 10 min in an ice water bath. The sample was then centrifuged at 12,000 rpm for 3 min (4 °C). Quantities of 120 μL aliquots of supernatant were transferred for LC-MS analysis. The sample extracts were analyzed using an LC-ESI-MS/MS system (UPLC, ExionLC AD, SCIEX Corporation, Shanghai/China; MS, QTRAP^®^ System, SCIEX Corporation, Shanghai/China). The analytical conditions were as follows: UPLC—column, Waters ACQUITY, Milford, MA, USA, UPLC HSS T3 C18 (1.8 µm, 2.1 mm × 100 mm); column temperature—40 °C; flow rate—0.4 mL/min; injection volume—2 μL; solvent system—water (0.1% formic acid) and acetonitrile (0.1% formic acid); solvent B gradient program—5% to 20% in 2 min, increased to 60% in the following 3 min, increased to 99% in 1 min, and held for 1.5 min, then brought back to 5% within 0.1 min, and held for 2.4 min. LIT and triple-quadrupole (SCIEX Corporation, Shanghai/China) scans were acquired on a triple-quadrupole–linear ion trap mass spectrometer (QTRAP, SCIEX Corporation, Shanghai/China), QTRAP^®^ LC-MS/MS System, SCIEX, Framingham, MA, USA, equipped with an ESI Turbo Ion-Spray interface, operating in positive and negative ion mode and controlled by Analyst 1.6.3 software (SCIEX Corporation, Shanghai/China). The ESI source operation parameters were as follows: source temperature 500 °C; ion spray voltage (IS) 5500 V (positive), −4500 V (negative); ion source gas I (GSI), gas II (GSII), curtain gas (CUR) was set at 55, 60, and 25.0 psi, respectively; the collision gas (CAD) was high. Instrument tuning and mass calibration were performed with 10 and 100 μmol/L polypropylene glycol solutions in QQQ and LIT modes, respectively. A specific set of MRM transitions was monitored for each period according to the metabolites eluted within this period.

Unsupervised PCA (principal component analysis) was performed by statistics function prcomp withinR (R Foundation for Statistical Computing, Vienna, Austria, www.r-project.org). The data were unit variance-scaled before unsupervised PCA. The HCA (hierarchical cluster analysis) results of samples and metabolites were presented as heatmaps with dendrograms, while Pearson correlation coefficients (PCC) between samples were calculated by the cor function in R and presented as only heatmaps. Both HCA and PCC were carried out by R package Complex Heatmap. For HCA, normalized signal intensities of metabolites (unit variance scaling) are visualized as a color spectrum. For two-group analysis, differential metabolites were determined by VIP (VIP > 1) and *p*-value (*p*-value < 0.05, Student’s *t* test). For multi-group analysis, differential metabolites were determined by VIP (VIP > 1) and *p*-value (*p*-value < 0.05, ANOVA). VIP values were extracted from the OPLS-DA result, which also contain score plots and permutation plots, generated using R package MetaboAnalystR, version 4.0. The data were log transform (log2) and mean centering before OPLS-DA. To avoid overfitting, a permutation test (200 permutations) was performed. Identified metabolites were annotated using KEGG Compound database (Kanehisa Laboratories, Kyoto, Japan; http://www.kegg.jp/kegg/compound/, accessed on 10 October 2024), annotated metabolites were then mapped to KEGG Pathway database (http://www.kegg.jp/kegg/pathway.html, accessed on 10 October 2024). Pathways with significantly regulated metabolites mapped to were then fed into MSEA (metabolite sets enrichment analysis), their significance was determined by hypergeometric test’s *p*-values.

### 2.6. Microbiome Sample Processing and Sequencing

After slaughter, rumen fluid from Xizang sheep was stored in sterile freezer tubes and kept at −80 °C. The rumen fluid was then used to extract DNA from the sheep. DNA extraction, primer design and synthesis, PCR amplification, and high-throughput sequencing of the contents were performed with the assistance of Metware (Wuhan, China). Total genome DNA was extracted from the rumen fluid of SFWS using the CTAB method. The concentration and purity of DNA were measured on a 1% agarose gel. Based on the concentration, DNA was diluted to 1 ng/L using sterile water. The PCR procedures included 15 L of Phusion High-Fidelity PCR Master Mix (New England Biolabs, Ipswich, MA, USA), 2 mM of forward and reverse primers, and around 10 ng of template DNA. A one-minute initial denaturation at 98 °C was followed by thirty cycles of ten seconds at 98 °C denaturation, thirty seconds of annealing at 50 °C, thirty seconds of elongation at 72 °C throughout the thermal cycling process, and, lastly, five minutes at 72 °C. We combined the PCR products with an equal volume of 1× loading buffer that contains SYB green and then ran the electrophoresis on a 2% agarose gel to detect the results. Equidensity ratios were used to combine the PCR products. A mixture of PCR products was then purified using a Qiagen Gel Extraction Kit (Qiagen, Frankfurt /Germany). Following the creation of sequencing libraries using the TruSeq DNA PCR-Free Sample Preparation Kit (Illumina, San Diego, CA, USA) in line with the manufacturer’s instructions, index codes were added. The library’s quality was assessed using the Agilent Bioanalyzer 2100 instrument and the Qubit@ 2.0 Fluorometer (Thermo Scientific, Waltham, MA, USA). After this, the library was sequenced using an Illumina NovaSeq machine, Illumina, Inc., San Diego, CA, USA, producing 250 bp paired-end reads^®^.

### 2.7. Sequencing Data Processing and Analysis

The raw reads were filtered using fastp (v0.22.0, https://github.com/OpenGene/fastp, accessed on 10 October 2024) to obtain high-quality reads; spliced using FLASH (v1.2.11, http://ccb.jhu.edu/software/FLASH/, accessed on 10 October 2024) to obtain the tag sequences, which were compared with the species annotation database by vsearch (v2.22.1, https://github.com/torognes/vsearch/, accessed on 10 October 2024) to detect chimeric sequences; and the chimeric sequences were finally removed to obtain the final effective data (effective tags). Effective sequence alignment using QIIME software (Uparse v7.0.1001), using the SILVA database, was clustered into operational taxonomic units (ASVs) with a sequence similarity threshold of 97%. Finally, the data of each sample were homogenized. Observed_otus/ASV, Chao1, Shannon, Simpson, ace, Goods-coverage, and PD_whole_tree indices were computed using the phyloseq (v1.40.0) [16] and vegan (v2.6.2) packages of R software (v4.2.0). The dilution curves, rank abundance curves, and species accumulation curves were plotted using R software (v4.2.0), and the differences between groups of alpha diversity index were also analyzed using R software; Unifrac distances were computed using the phyloseq (v1.40.0) package of R software (v4.2.0), and the clustering tree of UPGMA samples was constructed. The PCA, PCoA, and NMDS plots were plotted using R software [17] (v4.2.0). PCA analysis was performed using the stats package of R software, and PCoA and NMDS analyses were performed using the phyloseq (v1.40.0) package of R. The Tukey and Kruskal–Wallis tests were used to analyze the differences between groups in alpha diversity index. The Tukey test and Kruskal–Wallis test were used for parametric and nonparametric tests, respectively.

### 2.8. Data Analysis

Statistical analyses of body weight and serum data were performed using IBM SPSS statistical software (version 26.0) and graphs were produced using GraphPad Prism software (version 9.5). After testing the normality of data distribution, group comparisons were made by independent samples t-test and multiple comparisons by analysis of variance (ANOVA). The level of statistical significance was set at *p* < 0.05 to indicate statistical differences, while a higher level of significance was set at *p* < 0.01 to indicate high statistical significance.

### 2.9. Relevance Analysis

The principal differential microorganisms and metabolites were chosen for correlation analysis. Spearman correlation coefficients for microorganisms and metabolites were computed. Heatmaps were utilized to illustrate the correlation between distinct microorganisms and metabolites. Heatmaps were generated employing the stats package in R software.

## 3. Results

### 3.1. Effect of Different Castration Methods on Serum Testosterone and Body Weight in Male Xizang Sheep

**Figure 1:**
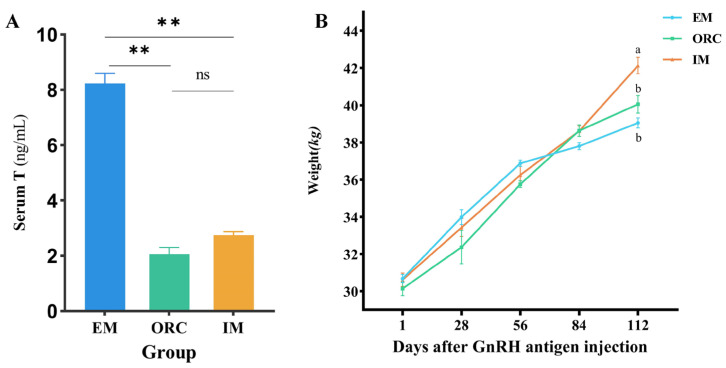
Changes in serum testosterone and body weight in three groups of male Xizang sheep (5 sheep in each group). (**A**) Serum testosterone levels in three groups of male Xizang sheep on day 112 of the official trial period. (**B**) Changes in body weights of male Xizang sheep in the three groups during the formal test period (day 1 was the time of the first GnRH vaccination in the IM group and day 56 was the time of the second GnRH vaccination in the IM group). ** denotes highly significant difference; ns denotes non-significant difference; different letters denote significant difference (*p* < 0.05); the same letter denotes non-significant difference (*p* > 0.05).

The data on serum testosterone levels in the three groups of male Xizang sheep showed that on day 112 of the formal trial, the serum testosterone levels in the ORC and IM groups were significantly lower than those in the EM group (*p* < 0.01), while there was no significant difference between the ORC and IM groups (*p* > 0.05). This indicates that both the surgical castration and GnRH immunoinjection methods used in this study were effective in suppressing serum testosterone levels and achieving the desired castration effect. Analysis of the body weights of five male Xizang sheep selected from each of the three experimental groups during the experimental cycle showed (Figure 1B) that the ORC group showed a slower increase in body weight during the first 56 days of the official experimental period, while the IM group showed a faster increase in body weight throughout the official experimental cycle. The final body weights of the IM group were significantly higher than those of the EM group and the ORC group (*p* < 0.05). Therefore, based on the purpose of the experiment, changes in body weight and serum, and other constraints, the EM and IM groups were selected for comparative analyses in the subsequent study.

### 3.2. Effects of GnRH Immunization on Rumen Microorganisms in Male Xizang Sheep

**Figure 2:**
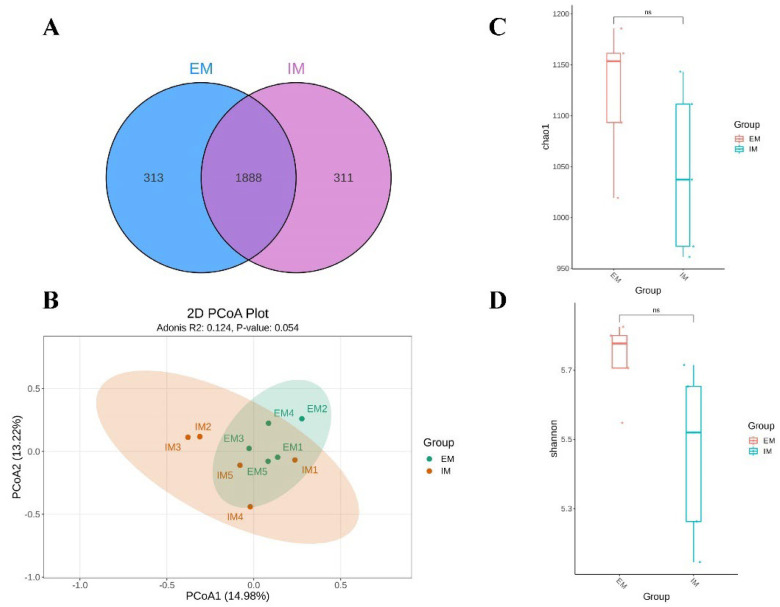
Effect of GnRH immunization on rumen microbial diversity in male Xizang sheep (EM VS IM, 5 sheep in each group). (**A**) ASV analysis of microorganisms. (**B**) Principal component analysis (PCoA) of microorganisms. (**C**) Chao1 exponential analysis of microorganisms (ns indicates that the difference is not significant). (**D**) Shannon exponential analysis of microorganisms (ns indicates that the difference is not significant).

Based on the experimental objectives of this study and data on serum testosterone and body weight, rumen fluid samples from male Xizang sheep EM and IM groups were selected for amplification of 16S rRNA sequences. After quality control of the sequencing data, a total of 2512 ASVs were obtained, of which 313 and 311 ASVs were unique in the EM and IM groups, respectively, and were examined by Venn diagram (Figure 2A). The ɑ diversity and β diversity of rumen microorganisms were assessed to explore the changes in rumen microbiota composition between the two groups. Similarly, the ɑ and β diversity of rumen microbes were analyzed to investigate the differences in microbial composition between the two groups of male Xizang sheep rumen. The principal concerted analysis (PCoA) of microorganisms showed (Figure 2B) that there was no substantial difference (*p* > 0.05) in β diversity between the two groups of male Xizang sheep samples. Meanwhile, the assessment of alpha diversity showed no significant difference (*p* > 0.05) in Chao 1 index (Figure 2C) or Shannon index (Figure 2D) between EM and IM groups. The above results indicated that immunocastration using GnRH had less effect on the diversity and abundance of the rumen microbiota of male Xizang sheep compared to the control group, while successfully achieving the desired castration effect.

**Figure 3:**
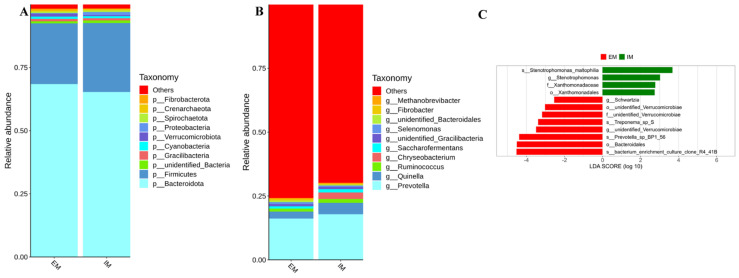
Effect of GnRH immunocastration on rumen microbial composition in male Xizang sheep (EM VS IM, 5 sheep in each group). (**A**) Percentage of rumen microbial abundance at portal level. (**B**) Percentage of rumen microbial abundance at genus level. (**C**) Analysis of rumen microbial LEfSe (LDA effect size).

To further elucidate the impact of GnRH immunocastration on the rumen microbiota of male Xizang sheep, the top 10 species with the highest abundance at the phylum and genus taxonomic levels were selected in each group for generating species relative abundance stacked plots based on species annotation results, aiming to visually represent species with elevated relative abundance and their proportions at diverse taxonomic levels in each group. At the phylum level (Figure 3A), the composition of rumen microbial communities exhibited a resemblance between the two groups, where *Firmicutes* and *Bacteroidota* emerged as the predominant phyla; however, the relative abundance of *Bacteroidota* in the IM group showed a 3.12% increase compared to that in the EM group, while the *Firmicutes* exhibited a 3.26% decrease relative to the EM group. By calculating the ratio of *Firmicutes* to *Bacteroidota*, it was found that the IM group was greater than the EM group. Furthermore, the relative abundance of the *Proteobacteria* clade was 0.9% higher in the IM group than in the EM group, whereas the *Verrucomicrobiota* clade displayed a 0.67% decrease compared to the EM group. At the genus level (Figure 3B), the most abundant genera in both groups were *Prevotella* and *Quinella*; nonetheless, the percentages of both *Prevotella* and *Quinella* were higher in the IM group than in the EM group.

In the current experiment, an LDA effect size (LEfSe) analysis was performed using LDA effect size values and *p*-values (LDA score *≥* 2, *p* < 0.05) to identify differential species. The results revealed (Figure 3C) that there were significantly different microbial communities in both groups of male Xizang sheep included in this study. In the IM group, the abundance of microbial communities such as *Stenotrophomonas_maltophilia*, *Stenotrophomonas*, and *Xanthomona* were significantly higher compared to the EM group. On the other hand, in the EM group, the abundance of microbial communities including *Bacteroidales*, *Prevotella_sp_BP1_56*, *unidentified_Verrucomicrobiae*, and *Schwartzia* were significantly higher than in the IM group.

### 3.3. Effect of GnRH Immunocastration on Rumen Metabolomics in Male Xizang Sheep

**Figure 4:**
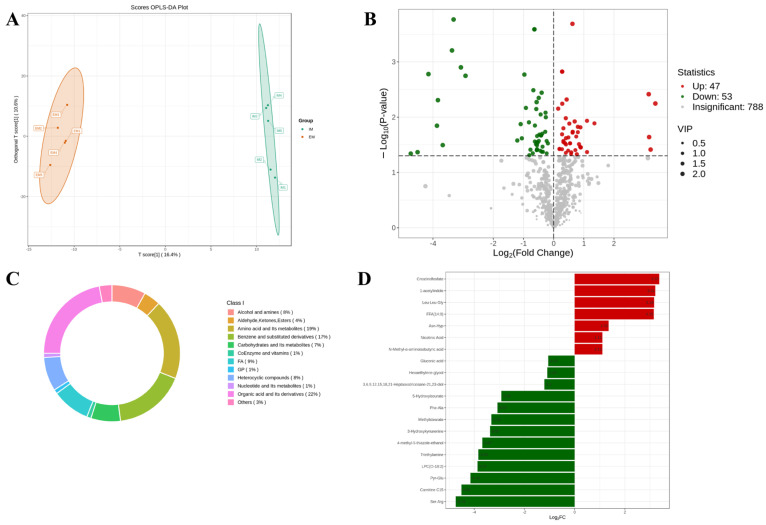
Effect of GnRH immunocastration on metabolite composition of rumen fluid in male Xizang sheep (EM VS IM, 5 sheep in each group). (**A**) OPLS-DA analysis of rumen metabolome. (**B**) volcano plot analysis of rumen metabolome. (**C**) Ring plot of rumen differential metabolite class composition. (**D**) Bar plot of rumen metabolite differential multiplicity.

In this study, a total of 10 rumen fluid samples from the EM and IM groups were subjected to metabolomics analysis, revealing the detection of 892 metabolites. The OPLS-DA analysis of the comprehensive metabolites (Figure 4A) distinctly discriminated between the metabolites of the EM and IM groups, suggesting that GnRH immunocastration induced perturbations in the rumen fluid metabolism of male Xizang sheep. The volcano plot analysis revealed (Figure 4B) the upregulation of 47 metabolites and the downregulation of 53 metabolites in the IM group. Table S1 displays all the differential metabolites (Table S1). The differential metabolites predominantly fell into 11 substance-level classifications (Figure 4C), comprising 22% organic acids and their derivatives, 19% amino acids and their metabolites, and 7% carbohydrates and their metabolites. Notably, creatinolfosfate, Leu-Leu-Gly, FFA (14:0), nicotinic acid, and N-Methyl-ɑ-aminoisobutyric acid exhibited significant upregulation in the IM group, whereas carnitine C15, 3-Hydroxykynurenine, methylstearate, and 5-Hydroxyisourate demonstrated marked downregulation in the IM group (Figure 4D).

**Figure 5:**
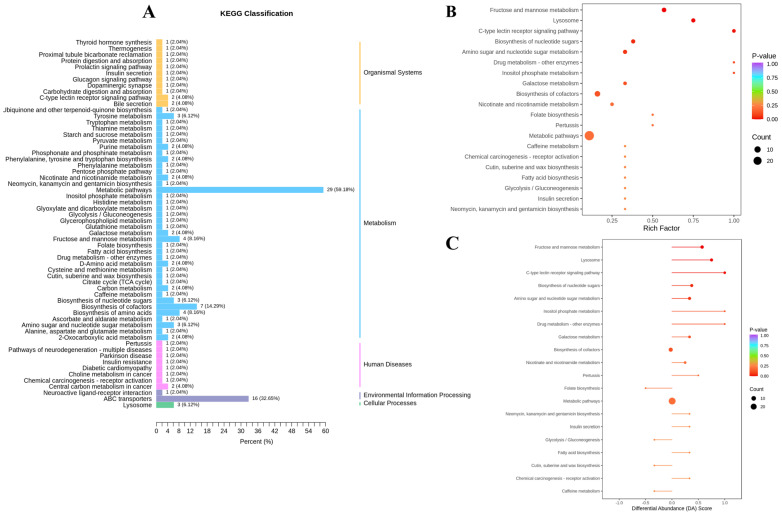
KEGG enrichment analysis of rumen fluid differential metabolites. (**A**) Differential metabolite pathway categorization diagram. (**B**) Differential metabolite pathway enrichment plot. (**C**) Differential metabolite abundance score plot.

The KEGG annotation results of all differential metabolites indicated their enrichment in 60 metabolic pathways in both groups. Among the top five pathways with the highest number of enriched metabolites, excluding the metabolic pathways, were ABC transporters (32.65%), biosynthesis of cofactors (14.29%), fructose and mannose metabolism (8.16%), biosynthesis of amino acids (8.16%), and amino sugar and nucleotide sugar metabolism (6.12%) (Figure 5A). Following the analysis of differential metabolites, KEGG pathway enrichment analysis was conducted, selecting and presenting the top 20 pathways ranked by *p*-value in ascending order. This analysis revealed that metabolic pathways exhibiting higher enrichment levels in the IM group included the C-type lectin receptor signaling pathway; drug metabolism—other enzymes; fructose and mannose metabolism; and lysosome (Figure 5B). Evaluation of the comprehensive metabolic alterations in the top 20 pathways, ranked by *p*-value, demonstrated that the IM group exhibited significant upregulation of the C-type lectin receptor signaling pathway; drug metabolism—other enzymes; fructose and mannose metabolism; and lysosome compared to the EM group (Figure 5C).

### 3.4. Metabolome–Microbiome Correlation Analysis

**Figure 6:**
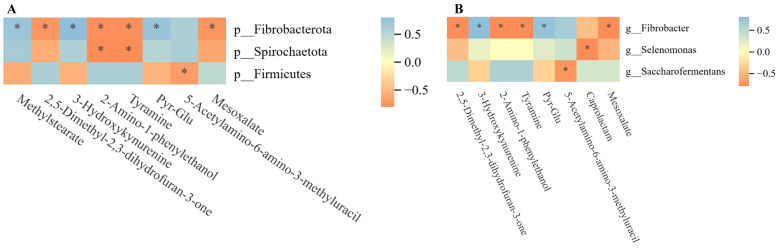
Association analysis: (**A**) Association analysis of top 10 phylum level microbiota with top 20 differential metabolites. (**B**) Association analysis of top 10 genus level microbiota with top 20 differential metabolites. * Significant correlations are indicated.

To ascertain the potential microbiological linkage of the alterations in rumen metabolites in male Xizang sheep subsequent to GnRH immunocastration, rumen microbes were meticulously examined through correlation analysis in relation to differential metabolites present in the rumen fluid. The results of the correlation analysis between the top 10 microbiota in gate-level abundance and the top 20 differential metabolites, as screened by their *p*-values, revealed a significant and positive correlation between the abundance of *Firmicutes* microbiota and 5-Acetylamino-6-amino-3-methyluracil. Similarly, the abundance of *Fibrobacterota* microbiota exhibited a significant positive correlation with methylstearate and tyramine, while displaying a negative correlation with methylstearate, 3-Hydroxykynurenine, and Pyr-Glu (Figure 6A). The results from the correlation analysis between the top 10 genera ranked by abundance and the top 20 differential metabolites, as determined by the *p*-value, highlighted the significant positive correlation between colony *Selenomonas* and the metabolite caprolactam, as well as the notable positive correlation of colony *Fibrobacter* with methylstearate and tyramine, while also showing a significant negative correlation with 3-Hydroxykynurenine (Figure 6B).

## 4. Discussion

Testosterone plays a key role in androgenic physiological functions, such as promoting the development of reproductive organs and stimulating sexual behavior [18,19]. Research has demonstrated that decreased testosterone levels heighten the susceptibility to obesity in males by influencing fat deposition [20]. Additionally, castration itself has been noted to stimulate the progression of obesity [21]. In the current investigation, both surgical castration and GnRH immunocastration markedly decreased serum testosterone levels in male Xizang sheep, aligning with previous related research findings [22,23]. This further corroborates that the implemented castration techniques in this study achieved the intended objectives and warrant continued exploration. It has been found that the growth rate of immunodenuded Bos indicus bulls was higher as compared to surgically denuded Bos indicus bulls [24]. The average daily weight gain of immunodenuded rams was higher than that of surgically denuded rams [25]. The current investigation revealed that the IM cohort exhibited accelerated weight gain and greater final weight compared to the EM group throughout the experimental period; meanwhile, the ORC group displayed a superior final weight to the EM group, indicating the influence of castration on the body weight increase in male Xizang sheep, with a more noticeable impact observed through immunocastration, albeit concurrently achieving the intended castration effect. Consequently, this outcome diverged from the findings of Cui S’s study on sheep [26], likely attributed to disparities not only in the experimental locale but also in the sheep breeds and feeding practices.

The establishment of rumen microorganisms in ruminants’ post-birth is widely acknowledged to be primarily dictated by maternal influence, subsequently evolving in response to factors encompassing diet, age, sex, and breed [27]. Research has shown that male and female mice harbor distinct microbiota compositions, a disparity that can be modified following male castration [28]. Consequently, a meticulous investigation was carried out on the microorganisms and metabolites present in the rumen fluid of GnRH-immunocastrated male Xizang sheep. Analysis performed on the chao1 index and Shannon index following the attainment of the requisite sequencing depth for both sample groups revealed that GnRH immunocastration had no substantial impact on the structural makeup of rumen microorganisms in male Xizang sheep, despite achieving the desired castration outcome. This observation led to speculation that the robust adaptability of male Xizang sheep, a breed accustomed to the plateau environment, may contribute to the relative stability of their rumen microbial population [29]. Furthermore, the grazing habits of male Xizang sheep in the plateau region, emphasizing free-range foraging, along with uncomplicated feeding practices, could potentially aid in preserving the stability of rumen microorganisms [30]. Furthermore, it is known that GnRH castration primarily influences the reproductive system of the animal [31], suggesting minimal impact on the holistic arrangement of rumen microbes in male Xizang sheep; however, comprehensive investigations are required to delve into the specific underlying causes.

The microbial community thriving in a healthy rumen is distinguished by the prevailing presence of *Bacteroidota* and *Firmicutes* [32]. *Bacteroidota* is associated with a substantial expression of genes encoding carbohydrate-active enzymes that facilitate the degradation of structural polysaccharides within the rumen [33]. Additionally, these organisms are involved in amino acid fermentation resulting in acetate production and participate in starch degradation, facilitating carbohydrate digestion and absorption [34]. The phylum *Firmicutes* primarily encompasses a variety of genera containing fibrolytic and cellulolytic *bacteria*, playing crucial roles in fiber breakdown and fat accumulation within the rumen [34,35]. A rise in the *Firmicutes/Bacteroidota* ratio could signify enhanced feed utilization by the animals [33]. Additionally, Wang et al. [36] proposed that enhancing the intestinal ratio of *Firmicutes* to *Bacteroidota* could potentially enhance the host’s ability to extract and store more energy from the diet. In this investigation, it was observed that *Firmicutes* and *Bacteroidota* were predominant in all sample sets from both experimental groups. However, it was noted that immunocastration led to a reduction in the proportion of *Bacteroidota* while enhancing the prevalence of Firmicutes. This observation suggests that GnRH immunocastration could potentially augment rumen fiber digestion and decomposition in male Xizang sheep. Furthermore, the *Firmicutes/Bacteroidota* ratio in the IM group surpassed that of the EM group, leading to the hypothesis that GnRH immunocastration could possibly ameliorate the digestive efficacy of foraged feeds in male Xizang sheep. At the genus level, *Prevotella* and *Quinella* were identified as the most abundant genera in both groups; however, the proportions of *Prevotella* and *Quinella* were higher in the IM group compared to the EM group. Research by Chen et al. [37] revealed that *Prevotella* is significantly linked to fat accumulation in pigs, with certain members of *Prevotella* producing short-chain fatty acids. Moreover, *Prevotella* has been shown to aid in the breakdown of dietary proteins and carbohydrates, offering potential benefits for glucose homeostasis and host metabolism [38]. The dominance of *Prevotella* in the intestinal microbiota has been linked to various animal traits such as feed intake, feeding efficiency, weight gain, and diarrhea incidence. This genus plays a crucial role in enhancing growth performance and mitigating intestinal stress and injury [39]. The intestinal microbiota driven by *Prevotella* has been linked to various animal traits, such as feed intake, feeding efficiency, weight gain, and the incidence of diarrhea, and is pivotal in enhancing growth performance while decreasing intestinal stress and injury [40]. The quantity of Quinella is significantly linked to the levels of carbohydrate metabolites [41], and the existence of genes encoding glucose, sorbitol, fructose, maltose, and mannose within g__Quinella indicates the capability of microbial members in this genus to break down polysaccharides to liberate smaller carbohydrates. The genome of Quinella also harbors the L-lactate dehydrogenase gene, indicating its ability to produce lactic acid or utilize it as a substrate for the formation of other small molecule compounds [42]. Therefore, increasing the proportion of readily fermentable carbohydrates in the rumen contents may affect the efficiency of rumen utilisation of feed. In conjunction with the preceding discussion, the greater abundance of and in the IM group compared to the EM group implies that castration could enhance the efficiency of digesting and catabolizing food in the rumen. This may particularly impact the capacity to digest and absorb proteins, carbohydrates, and fats by influencing the rumen microbiota of male Xizang sheep, ultimately expediting the fattening and weight gain of castrated male Xizang sheep.

The rumen fluid metabolome data from this study indicate that immunocastration influenced metabolite levels in the rumen. Despite the limited literature on the impact of castration on rumen metabolism in sheep, our findings align with research conducted by Whon TW et al. [22] and Shi J et al. [43], focusing on variations in intestinal metabolite concentrations in castrated cattle. Carbohydrate metabolism is paramount in governing animal health, predominantly arising from the breakdown of starch and cellulose by microbial enzymes within the rumen to yield glucose. Subsequently, glucose proceeds through the glycolytic pathway, ultimately generating pyruvic acid—a key precursor for volatile fatty acid biosynthesis [44]. Functioning as metabolites of carbohydrates, volatile fatty acids play a pivotal role in governing rumen epithelial differentiation, apoptosis, and morphology [45]. Sutton et al. [46] identified that carbohydrate end products serve as energy substrates, inflammatory modulators, and signaling molecules within the rumen. Within the scope of this investigation, D-glucose-6-phosphate, D-mannose 6-phosphate, fructose-6-phosphate, fructose, mannose, and myo-inositol—comprising a collective of six carbohydrates and their respective metabolites—displayed notable upregulation in the IM group, exhibiting distinct differences in metabolite levels compared between the EM and IM cohorts. The presence of d-Glucose 6-phosphate has the potential to accelerate glycolysis by modulating the starch and sucrose metabolic pathways, subsequently impacting meat quality outcomes [47]. Interestingly, in contrast, the findings by Zhang et al. [48] demonstrated that the accumulation of d-glucose 6-phosphate led to a suppression of hexokinase activity, inhibition of glycolysis, and a deceleration in the rate of pH decline. Changes in the concentrations of d-glucose 6-phosphate and d-fructose 6-phosphate may be associated with the pentose phosphate metabolic pathway, which is predominantly active in the rumen epithelium and serves as the primary route for synthesizing ribose 5-phosphate. This compound, ribose 5-phosphate, plays a crucial role in facilitating cell growth and proliferation [49]. Mannose serves as a prominent monomer, and its upregulation has the potential to impact the synthesis of glycoproteins. Substrates for glycoprotein biosynthesis [50] and upregulation of mannose may affect glycoprotein synthesis. Accordingly, the findings of this study suggest that GnRH-immunocastration can enhance the digestion and absorption of carbohydrates from feed in the rumen of male Xizang sheep. This enhancement leads to the increased production of metabolites, including volatile fatty acids and glucose, potentially improving meat quality, yield, pH levels of ruminal fluid, and overall ruminal health.

Approximately 50–85% of short-chain fatty acids in the rumen of ruminants are absorbed directly through the rumen epithelium [51]. Beyond its pivotal function in nutrient uptake, the rumen epithelium plays a crucial role in establishing a barrier against potentially harmful substances within the rumen [52,53]. Moreover, ensuring the efficient transportation of fermentation byproducts—such as short-chain fatty acids—by the rumen epithelium is paramount in averting acidosis, safeguarding the rumen epithelium from damage, and upholding rumen homeostasis [51]. Thus, the preservation of the rumen epithelial microenvironment and the integrity of the rumen epithelium are imperative for the maintenance of ruminant health and productivity. It has been proposed that the accumulation of organic acids may elevate the susceptibility to subacute rumen acidosis in dairy cows [54]. Comparative analysis of rumen samples with significant metabolites in the IM and EM groups of this study unveiled a downregulation of 14 organic acids and their derivatives in the IM group. Notably, compounds such as 5-Hydroxyisourate and 3Hydroxyisourate, among others, are capable of being ultimately catabolized to urea or ammonia through the synergistic action of enzymes and microorganisms [55]. The excess of nitrogen sources, including urea, could potentially incite inflammation and ulceration in rumen tissues. Furthermore, this investigation identified an upregulation of the metabolites nicotinic acid and tyramine in the IM group through metabolite analysis of both groups. It has been documented that microbial processes within the rumen are responsible for the synthesis of nicotinic acid, serving as a primary source of niacin in ruminants. Nicotinic acid, a form of vitamin B3, has been verified to confer a protective effect on the rumen epithelium. Additionally, nicotinamide could enhance mucus secretion in the gastric mucosa, fostering the repair and regeneration of surface cells, thus mitigating gastric mucosal injury [56,57]. Furthermore, niacinamide exhibits antioxidant and anti-inflammatory properties, which can alleviate oxidative stress and inflammation within the rumen, subsequently minimizing damage to the rumen epithelium [58,59]. Tyramine, a byproduct of tyrosine metabolism, is classified as a biogenic amine [60]; while low concentrations of biogenic amines are vital for normal cellular growth and differentiation, elevated levels can impede epithelial regeneration and provoke epithelial harm [61]. Tyramine is a commonplace physiological constituent in plants, animals, and many microorganisms, playing crucial roles in cellular physiology. Studies have indicated that heightened levels of biogenic amines in animals are intricately linked to rumen acidosis [62]. Furthermore, KEGG-based annotation revealed a significant enrichment in signaling pathways associated with immunity and inflammation in the IM group, with a general trend towards upregulation in pathway expression, including the C-type lectin receptor signaling pathway and drug metabolism—other enzymes. The C-type lectin receptor is known to recognize endogenous and exogenous ligands and is considered the predominant family of pattern recognition receptors in detecting fungi, thus playing a vital role in targeting antifungal innate and adaptive immunity [63]. Therefore, based on the findings of this study, which indicated significant downregulation of 14 organic acids and significant upregulation of nicotinic acid and tyramine in the IM group, it can be postulated that GnRH immunocastration exerts a beneficial effect on maintaining intra-ruminal homeostasis and the health of ruminal epithelial cells in male Xizang sheep.

## 5. Conclusions

The results of this study showed that the use of the GnRH immunocastration technique increased the rate of weight gain in Xizang sheep while achieving castration. Secondly, rumen microbiome analysis showed that the GnRH immunocastration technique increased the ratio of *Firmicutes/Bacteroidetes* at the phylum level and the abundance of *Prevotella* and *Quinella* bacteria at the genus level, and it was hypothesized that the GnRH immunocastration technique could improve the efficiency of food digestion and catabolism in the rumen. Metabolomic analysis of rumen fluid showed that the metabolites 5-hydroxyisolate and 3-hydroxyisolate were significantly downregulated and niacin and tyramine were significantly upregulated in rumen fluid after GnRH immunocastration, which was speculated to have a positive effect on the maintenance of rumen endo-environmental homeostasis and the health of rumen epithelial cells in Xizang sheep. This study provides further support for the application and promotion of GnRH immunocastration technology.

## Data Availability

All data in this study are available upon request by contact with the corresponding author.

## Supplementary Materials

The following supporting information can be downloaded at: https://www.mdpi.com/article/10.3390/ani14202942/s1, Table S1: Ruminal differential metabolites in Male Xizang Sheep after GnRH-immunized castration. Differential metabolites listed in the table are *p*-value < 0.05, VIP value > 1.
