# Supplementary material for: GnRH Immunocastration in Male Xizang Sheep: Impacts on Rumen Microbiome and Metabolite Profiles for Enhanced Health and Productivity
Source: Animals (Basel). 2024 Oct 11;14(20):2942. doi: 10.3390/ani14202942 (PMC11503817; doi:10.3390/ani14202942)
Supplement: Supplementary file 1 [file animals-14-02942-s001.zip › animals-3210666-supplementary.pdf]

Supplementary

Table S1. Ruminal differential metabolites in Male Xizang Sheep after GnRH-immunized castration.

Differential metabolites listed in the table are P-value<0.05, VIP value>1.

Compounds	Class I	VIP	P-value	Fold_Change	Log2FC	Type
D-Glucose 6-Phosphate	Carbohydrates and Its metabolites	1.835	0.0352	1.862	0.897	up
D-Mannose6phosphate	Carbohydrates and Its metabolites	1.835	0.0352	1.862	0.897	up
Fructose	Carbohydrates and Its metabolites	1.661	0.0376	1.144	0.194	up
Fructose-6-phosphate	Carbohydrates and Its metabolites	1.835	0.0352	1.862	0.897	up
Gluconic acid	Carbohydrates and Its metabolites	1.961	0.0241	0.482	-1.053	down
Mannose	Carbohydrates and Its metabolites	1.661	0.0376	1.144	0.194	up
Myoinositol	Carbohydrates and Its metabolites	1.661	0.0376	1.144	0.194	up
Ala-Arg	Amino acid and Its metabolites	1.581	0.0282	0.681	-0.555	down
Ala-Pro	Amino acid and Its metabolites	1.742	0.0270	1.250	0.322	up
Arg-Thr	Amino acid and Its metabolites	1.831	0.0131	1.434	0.520	up
Asn-Hyp	Amino acid and Its metabolites	1.726	0.0129	2.551	1.351	up
Cys-Pro	Amino acid and Its metabolites	1.673	0.0232	1.403	0.488	up
DL-2-Methylglutamic acid	Amino acid and Its metabolites	2.043	0.0120	1.621	0.697	up
His-Pro	Amino acid and Its metabolites	1.709	0.0405	0.736	-0.442	down
Leu-Leu-Gly	Amino acid and Its metabolites	2.339	0.0229	8.963	3.164	up
Met-Thr	Amino acid and Its metabolites	1.648	0.0468	1.704	0.769	up
N-Methyl-α-aminoisobutyric acid	Amino acid and Its metabolites	1.782	0.0116	2.152	1.105	up
Nα-Acetyl-L-glutamine	Amino acid and Its metabolites	1.591	0.0224	1.681	0.749	up
Phe-Ala	Amino acid and Its metabolites	2.433	0.0013	0.119	-3.073	down
Phe-Val	Amino acid and Its metabolites	1.786	0.0190	1.544	0.627	up
Pro-Ala	Amino acid and Its metabolites	1.754	0.0204	1.214	0.280	up
Pyr-Glu	Amino acid and Its metabolites	2.447	0.0017	0.056	-4.149	down
S-Sulfo-L-Cysteine	Amino acid and Its metabolites	1.552	0.0343	1.834	0.875	up
S-methyl-L-thiocitrulline	Amino acid and Its metabolites	1.733	0.0296	1.298	0.376	up
Ser-Arg	Amino acid and Its metabolites	2.322	0.0456	0.038	-4.735	down
Val-Thr	Amino acid and Its metabolites	1.516	0.0442	1.323	0.404	up
3-(3-Methoxyphenyl)Propionic Acid	Organic acid and Its derivatives	1.675	0.0450	0.620	-0.690	down
3-Guanidinopropionic acid	Organic acid and Its derivatives	1.591	0.0380	1.212	0.277	up
3-Hydroxykynurenine	Organic acid and Its derivatives	2.445	0.0006	0.097	-3.368	down
5-Hydroxyisourate	Organic acid and Its derivatives	2.431	0.0018	0.132	-2.920	down
Allantoin	Organic acid and Its derivatives	1.928	0.0100	0.836	-0.259	down
Colneleic acid	Organic acid and Its derivatives	1.691	0.0378	0.683	-0.550	down
Creatinolfosfate	Organic acid and Its derivatives	2.409	0.0057	10.343	3.371	up
Dehydroascorbic acid	Organic acid and Its derivatives	1.615	0.0432	1.501	0.586	up
Ethylparaben	Organic acid and Its derivatives	1.679	0.0269	0.851	-0.232	down
Geranic acid	Organic acid and Its derivatives	1.732	0.0220	0.684	-0.548	down

Isonicotinic acid	Organic acid and Its derivatives	1.760	0.0308	1.790	0.840	up
Mesoxalate	Organic acid and Its derivatives	1.953	0.0057	1.219	0.286	up
Octadec-9-ene-1,18-dioic-acid	Organic acid and Its derivatives	1.840	0.0142	0.651	-0.620	down
Oxaloacetic acid	Organic acid and Its derivatives	2.034	0.0071	0.671	-0.575	down
Phenylpyruvic Acid	Organic acid and Its derivatives	1.882	0.0388	0.587	-0.769	down
Phosphonoacetic acid	Organic acid and Its derivatives	1.686	0.0276	0.649	-0.623	down
Piperonylic acid	Organic acid and Its derivatives	1.699	0.0311	1.327	0.408	up
Pyrazine-2-carboxylic acid	Organic acid and Its derivatives	1.733	0.0297	1.458	0.544	up
Salicylic acid	Organic acid and Its derivatives	1.840	0.0214	0.748	-0.418	down
Shikimic Acid	Organic acid and Its derivatives	2.285	0.0053	0.676	-0.564	down
Subericacid	Organic acid and Its derivatives	1.775	0.0268	0.712	-0.491	down
Uric acid	Organic acid and Its derivatives	1.874	0.0184	1.550	0.632	up
1-acetylindole	Heterocyclic compounds	2.341	0.0385	9.272	3.213	up
2,5-Dimethyl-2,3-dihydrofuran-3-one	Heterocyclic compounds	2.226	0.0002	1.540	0.623	up
5-hydroxyindole-2-carboxylic acid	Heterocyclic compounds	1.485	0.0462	1.493	0.578	up
Caprolactam	Heterocyclic compounds	2.088	0.0048	1.348	0.431	up
Imidazole-4-methanol	Heterocyclic compounds	1.867	0.0089	0.742	-0.431	down
Imidazoleacetic acid	Heterocyclic compounds	1.781	0.0184	0.825	-0.278	down
Pyrimidine-4-carboxylic acid	Heterocyclic compounds	1.733	0.0297	1.458	0.544	up
TriacetonaMine	Heterocyclic compounds	1.990	0.0068	0.529	-0.919	down
10-Hydroxystearic Acid	FA	1.669	0.0342	0.775	-0.367	down
13-methylmyristic acid	FA	1.771	0.0199	0.770	-0.377	down
16-Hydroxyhexadecanoic acid	FA	2.243	0.0003	0.643	-0.637	down
2-hydroxyhexadecanoic acid	FA	2.243	0.0003	0.643	-0.637	down
8,15-DiHETE	FA	1.869	0.0153	1.851	0.888	up
Azelaoyl PAF	FA	2.055	0.0033	0.628	-0.672	down
Carnitine C15	FA	2.381	0.0430	0.044	-4.509	down
FFA(14:0)	FA	2.415	0.0038	8.879	3.150	up
FFA(15:0)	FA	1.836	0.0159	1.252	0.324	up
1,4-DPCA	Benzene and substituted derivatives	1.875	0.0149	1.743	0.802	up
2',4',6'-Trihydroxyacetophenone	Benzene and substituted derivatives	1.710	0.0394	0.678	-0.560	down
2,6-Di-tert-butyl-4-(hydroxymethyl)phenol	Benzene and substituted derivatives	1.959	0.0070	1.110	0.151	up
2-Butyl-3-(4-hydroxybenzoyl)benzofuran	Benzene and substituted derivatives	1.687	0.0287	0.695	-0.525	down
3,4'-Dihydroxyflavonoid	Benzene and substituted derivatives	1.818	0.0298	0.876	-0.190	down
3,6,9,12,15,18,21-	Benzene and substituted derivatives	2.059	0.0264	0.434	-1.204	down

Heptaoxatricosane-21,23-diol						
3-Hydroxy-4-methoxybenzoic acid	Benzene and substituted derivatives	1.732	0.0423	0.781	-0.357	down
3-Methoxysalicylic Acid	Benzene and substituted derivatives	1.764	0.0322	0.683	-0.551	down
4-Hydroxybenzoic Acid	Benzene and substituted derivatives	1.840	0.0214	0.748	-0.418	down
5-Methoxysalicylic Acid	Benzene and substituted derivatives	1.791	0.0309	0.667	-0.585	down
Benzoic Acid	Benzene and substituted derivatives	1.617	0.0423	0.763	-0.391	down
Enterodiol	Benzene and substituted derivatives	1.633	0.0187	1.744	0.802	up
Methyl-Vanillate	Benzene and substituted derivatives	2.181	0.0045	0.710	-0.494	down
Orcinol	Benzene and substituted derivatives	1.717	0.0282	1.253	0.325	up
Tyramine	Benzene and substituted derivatives	2.098	0.0015	1.216	0.282	up
Tyrosol	Benzene and substituted derivatives	1.840	0.0214	0.748	-0.418	down
m-Cresol	Benzene and substituted derivatives	1.566	0.0451	0.852	-0.230	down
2-(α-D-mannosyl)-3-phosphate glyceride	Alcohol and amines	1.605	0.0394	1.647	0.720	up
2-Amino-1-phenylethanol	Alcohol and amines	2.098	0.0015	1.216	0.282	up
3-Hydroxybenzyl alcohol	Alcohol and amines	1.922	0.0124	0.567	-0.818	down
4-methyl-5-thiazole-ethanol	Alcohol and amines	2.309	0.0320	0.078	-3.675	down
Heptethylene-glycol	Alcohol and amines	2.202	0.0017	0.511	-0.970	down
Hexaethylene-glycol	Alcohol and amines	2.094	0.0134	0.469	-1.092	down
N,N′-dicyclohexylcarbodiimide	Alcohol and amines	1.874	0.0104	1.322	0.403	up
Tetraethylene-glycol	Alcohol and amines	1.749	0.0487	0.572	-0.807	down
Nicotinic Acid	CoEnzyme and vitamins	1.737	0.0428	2.153	1.107	up
LPC(O-18:2)	GP	2.373	0.0143	0.069	-3.866	down
5-Acetylamino-6-amino-3-methyluracil	Nucleotide and Its metabolites	2.182	0.0036	0.759	-0.397	down
Gemfibrozil	Others	1.592	0.0403	1.396	0.481	up
Orsellinate	Others	1.710	0.0394	0.678	-0.560	down
Triethylamine	Others	2.390	0.0049	0.070	-3.835	down
D-Erythronolactone	Aldehyde,Ketones,Esters	1.775	0.0240	1.327	0.408	up
Marmesin	Aldehyde,Ketones,Esters	1.717	0.0207	0.720	-0.474	down
Methylstearate	Aldehyde,Ketones,Esters	2.454	0.0002	0.100	-3.316	down
Monobutyl phthalate	Aldehyde,Ketones,Esters	1.876	0.0083	0.830	-0.268	down
